# Representation of the hierarchical and functional structure of an ambulatory network of medical consultations through Social Network Analysis, with an emphasis on the role of medical specialties

**DOI:** 10.1371/journal.pone.0290596

**Published:** 2024-02-15

**Authors:** Fernando Martín Biscione, Juliano Domingues da Silva

**Affiliations:** 1 Department of Data Science in Healthcare, Healthcare Superintendence, Unimed-Belo Horizonte Healthcare Plan, Belo Horizonte, Minas Gerais State, Brazil; 2 Department of Administration, Center for Socioeconomic Studies, State University of Maringá, Maringá, Paraná State, Brazil; Iuliu Hațieganu University of Medicine and Pharmacy: Universitatea de Medicina si Farmacie Iuliu Hatieganu, ROMANIA

## Abstract

**Background:**

Ambulatory Health Care Networks (Amb-HCN) are circuits of patient referral and counter-referral that emerge, explicitly or spontaneously, between doctors who provide care in their offices. Finding a meaningful analytical representation for the organic and hierarchical functioning of an Amb-HCN may have managerial and health policymaking implications. We aimed to characterize the structural and functional topology of an Amb-HCN of a private health insurance provider (PHIP) using objective metrics from graph theory.

**Methods:**

This is a cross-sectional quantitative study with a secondary data analysis study design. A Social Network Analysis (SNA) was conducted using office visits performed between April 1, 2021 and May 15, 2022, retrieved from secondary administrative claim databases from a PHIP in Belo Horizonte, Southeastern Brazil. Included were beneficiaries of a healthcare plan not restricting the location or physician caring for the patient. A directional and weighted network was constructed, where doctors were the vertices and patient referrals between doctors, within 7–45 days, were the network edges. Vertex-level SNA measures were calculated and grouped into three theoretical constructs: patient follow-up (aimed at assessing the doctor’s pattern of patient follow-up); relationship with authorities (which assessed whether the doctor is an authority or contributes to his or her colleague’s authority status); and centrality (aimed at positioning the doctor relative to the network graph). To characterize physician profiles within each dimension based on SNA metrics results, a K-means cluster analysis was conducted. The resulting physician clusters were assigned labels that sought to be representative of the observed values of the vertex metrics within the clusters.

**Findings:**

Overall, 666,263 individuals performed 3,863,222 office visits with 4,554 physicians. A total of 577 physicians (12.7%) had very low consultation productivity and contributed very little to the network (i.e., about 1.1% of all referrals made or received), being excluded from subsequent doctor profiles analysis. Cluster analysis found 951 (23.9%) doctors to be central in the graph and 1,258 (31.6%) to be peripheral; 883 (22.2%) to be authorities and 266 (6.7%) as seeking authorities; 3,684 (92.6%) mostly shared patients with colleagues, with patient follow-up intensities ranging from weak to strong. Wide profile dispersion was observed among specialties and, more interestingly, within specialties. Non-primary-care medical specialties (e.g., cardiology, endocrinology etc.) were associated with central profile in the graph, while surgical specialties predominated in the periphery, along with pediatrics. Only pediatrics was associated with strong and prevalent (i.e., low patient sharing pattern) follow-up. Many doctors from internal medicine and family medicine had unexpectedly weak and shared patient follow-up profiles. Doctor profiles exhibited pairwise relationships with each other and with the number of chronic comorbidities of the patients they treated. For example, physicians identified as authorities were frequently central and treated patients with more comorbidities. Ten medical communities were identified with clear territorial and specialty segregation.

**Conclusions:**

Viewing the Amb-HCN as a social network provided a topological and functional representation with potentially meaningful and actionable emerging insights into the most influential actors and specialties, functional hierarchies, factors that lead to self-constituted medical communities, and dispersion from expected patterns within medical specialties.

## Introduction

Health Care Networks (HCN) can be defined as the way in which health systems organize their health actions and services in an integrated, functional, and hierarchical manner, according to the different technological densities each one offers, to ensure care for the population being served [1]. The operational structure of HCN is therefore constituted by the different actors in health care (e.g., hospitals, doctor offices, diagnostic and therapy outpatient clinics, outpatient pharmacy, home care, etc.) and the connections that link them. Health Care Networks are characterized by the development of horizontal and vertical relationships between the various multi-professional care points for the patient in a more or less regulated manner. This network organizational arrangement of health systems is justified by fundamentals such as economies of scale, regionalization (or territorial coverage) of care, and guarantee of quality, sufficiency, and access to health for the population being assisted [1]. Another basic principle of HCN is that of structured levels of care according to technological density and rational use of resources, ranging from the level of lowest density (Primary Health Care) to intermediate density (Secondary Health Care), to the highest technological density level (Tertiary Health Care). It is the responsibility of Primary Health Care to be the first level of care, with a resolution function for the vast majority of the population’s health problems, from which specialized care is activated [1].

Regarding the ambulatory level of health care, this study will conceptualize the ambulatory care network (Amb-HCN) as the circuits of referral and counter-referral established, explicitly or spontaneously, between the doctors who attend to patients in their offices. In our setting, it is in the context of the doctor’s office where the primary level of health care takes place, with low technological density, and part of the secondary care (specialized consultations). Therefore, characterizing the organic and hierarchical functioning of a HCN through objective metrics can be strategically important for health managers, allowing them to: identify informal patterns or hierarchies among health actors that reveal the forces governing the organic functioning of the network; compare the observed structure of the HCN with the structure of other external networks, with the same network over time, or with that expected according to the health care model proposed by the manager for the network under his or her responsibility; identify actors with positive or negative influence on the HCN, according to the objectives defined by the manager; seek correlations between HCN performance metrics and attributes of health outcomes, quality or value delivered to users; propose reimbursement models based on results, quality and value in health. The ultimate goal is to provide information to health managers for setting policies that enable corrective or preventive decision-making, leading to continuous improvement of the network’s performance, quality, and efficiency with a patient-centered approach.

Because Amb-HCN involve manifold interconnected doctors with underlying roles and hierarchies–either formal or informal–unraveling the properties of an Amb-HCN is challenging. Moreover, as network behavior may arise from unknown, dynamic, or ill-defined factors, finding objective metrics that translate into meaningful healthcare outcomes or processes is a hard methodological task [2, 3]. Recent comprehensive literature reviews found that Social Network Analysis (SNA) has received strong interest from the scientific community for the study of numerous health phenomena that are inherently relational, complex, and dynamic, including, but not limited to, identifying relationships and personas, analyzing dissemination of innovations, and studying patterns of information exchange or collaboration among actors in diverse areas such as education, health promotion, infectious diseases spread, digital health, management, regulation, etc. [2, 3]. Social Network Analysis is a set of methods and concepts based on graph theory that analyze systems whose properties stem from the relationships between entities. The value of SNA in determining the properties of HCN has been scrutinized in recent studies. Researchers applied SNA to administrative claim data to reveal patient sharing patterns among physicians and found it to be an accurate method for finding hidden or informal referral networks of doctors [4] who exhibit close working relationships and manage to keep the vast majority of patient care within the networks [5]. By applying SNA to networks of professional teams who cared for diabetic patients, Ostovari et al. [6] identified key professionals and healthcare providers in the network. The same researchers found that when primary care physicians had high values in community-level centrality measures (i.e., closeness, betweenness, and degree), the diabetic, hypertensive, or dyslipidemic patients they cared for had lower hospitalization and emergency department visit rates [7]. Similar results were reported in another study, in which patients with cardiovascular diseases who were cared for by healthcare teams with dense interactions and low centralization had 38% fewer hospitalization days and lower healthcare costs compared to patients cared for by teams with less dense interactions that revolved around a few central professionals. Face-to-face, dense interactions among team members were also associated with more effective control of hypercholesterolemia and a 73% lower need for emergency department visits [8]. Although these and other studies point to promising results in the use of SNA for analyzing complex HCN, the most appropriate set of measures and evaluation metrics, as well as their clinical and administrative/managerial significance, remain uncertain [2, 9, 10].

The general purpose of this study was to represent the structural and functional topology of the Amb-HCN of a private health insurance provider (PHIP) through objective measures and metrics, based on the referral and counter-referral circuits, whether explicit or spontaneous, established between network physicians during patient care in their offices. As specific objectives, this study sought to: a) propose the use of doctors’ network measures, profiles and dimensions for the operational definition of Amb-HCN attributes considered important for the PHIP strategy; b) analyze these measures and metrics according to physicians’ specialties, seeking to determine their relative contribution to the Amb-HCN functioning; c) evaluate the relationship of doctors’ network measures with the number of chronic comorbidities of the patients they cared for; and d) identify self-organized communities of physicians, analyzing their coverage, territorial distribution, and specialty profiles.

## Materials and methods

### Study design

This is a cross-sectional exploratory and explanatory quantitative study with a secondary data analysis study design.

### Setting and period

The study was conducted on the beneficiaries base of a PHIP located in Belo Horizonte, capital city of Minas Gerais state, Southeastern Brazil. This company has a coverage area in Belo Horizonte and 33 other municipalities in its metropolitan region. As of April 2023, it provided assistance to more than 1.538 million beneficiaries (of whom over 80% are linked to group health insurance contracts financed by employers). Along with the offices of over 5,300 accredited primary and non-primary care physicians, the PHIP has a comprehensive range of outpatient services (e.g., oncology, renal replacement therapy, rehabilitation, home care, diagnostic imaging and laboratory, emergency care, etc.) and 20 hospital units, of which four are self-administered. Since 2021, it offers online consultations. The PHIP is widely recognized as a national market leader, according to objective and public rankings of governance and quality from regulatory and accreditation agencies.

The study considered a portfolio of 1,042,654 individuals who, between April 2021 and March 2022, were beneficiaries of a healthcare plan that did not restrict the location or physician who cares for the patient, remaining at his or her discretion and convenience. The study evaluated all office visits made by patients between April 1, 2021 and May 15, 2022.

### Data and definitions

The study’s database was extracted from secondary databases maintained by the PHIP in its own Data Warehouse and included the following data: physician identification; physician age and specialty; patient satisfaction score with doctor care, reported after the consultation, ranked on a scale of 0 (worst possible satisfaction) to 10 (best possible satisfaction); patient identification; office medical visits made by the patient, including location, physician who performed the consultation, and date of consultation; number of chronic comorbidities of the patients recorded in the PHIP, classified according to Elixhauser et al., Charlson-Deyo et al., and Feudtner et al. [11, 12]. All these data represent administrative claim data or beneficiary registration information routinely collected by the PHIP, that make up comprehensive and mature databases that undergo strict security, governance, and validation procedures.

The data reported in this research was retrieved in April 2023. However, programming codes and methods were developed for business–rather than research–purposes on a subset of the same dataset made available to the lead researcher (who serves as data science manager in the PHIP) in October 2022, in accordance with the PHIP’s institutional compliance and legal standards. The data was retrieved from data repositories at the individual level in a pseudonymized form and handled anonymously thereafter.

### Data analysis

**Fig 1:**
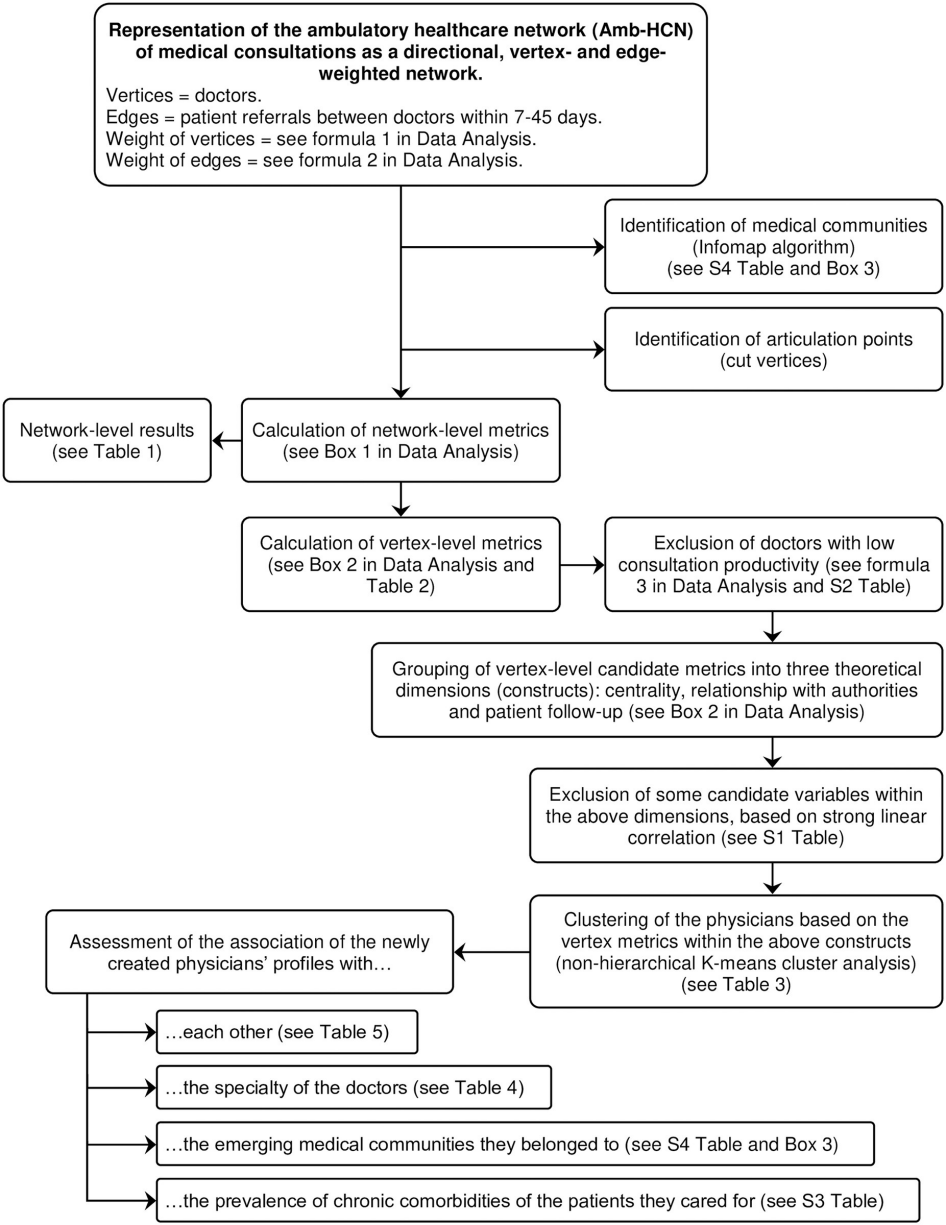
Summary flowchart of the methodological approach followed in this study.

The methodological approach outlined in this section is summarized step-by-step as a flowchart in Fig 1.

A Social Network Analysis was conducted to evaluate the properties of the Amb-HCN, conceptualizing the latter as the set of referral and counter-referral circuits of patients established between physicians, either explicitly or spontaneously, who assist patients in their offices. The SNA design followed the general principles recommended in Blanchet et al. [13] and De Brún et al. [14]. According to the classification proposed by Benhiba et al. [15], it is a structural SNA analysis (i.e., describing, at discrete intervals, the topology of the network, the roles of the vertices, describing communities and subgroups, etc.) with an egocentric view (i.e., characterizing actors according to the relationship they have with their immediate network). The constitutive elements of this network were as follows:

a) vertices (V): represented by the physicians v*i*… v*j* who performed the consultations in their offices;
b) edges (E): represented by patients who, after a consultation with a particular physician v*i*, had a consultation with another physician v*j* within an interval of 7 to 45 days, thus linking physician v*i* to physician v*j*. This time range was chosen because it represents the interval in which most referrals between professionals occur in our settings and would reveal referrals motivated by the same health-related problems;
c) vertex weight (Vw): since the contribution of each physician to the total number of consultations in the network depends on their own characteristics and, at the same time, the specialty to which they belong, the weight of the vertices was represented by the product below:
$$\begin{array}{ll} \text{Weight}\;\text{of}\;\text{vertex}\;\text{v}i & =\left(\frac{\begin{array}{c} \text{Total}\;\text{number}\;\text{of}\;\text{consultations}\;\text{by}\\ \text{physician}\;\text{v}i\;\text{in}\;\text{the}\;\text{period} \end{array}}{\begin{array}{c} \text{Total}\;\text{number}\;\text{of}\;\text{consultations}\;\text{by}\\ \text{physician}\;\text{v}i’\text{s}\;\text{specialty}\;\text{in}\;\text{the}\;\text{period}\; \end{array}}\;\right)\times \\ & \left(\frac{\begin{array}{c} \text{Total}\;\text{number}\;\text{of}\;\text{consultations}\;\text{by}\\ \text{physician}\;\text{v}i’\text{s}\;\text{specialty}\;\text{in}\;\text{the}\;\text{period} \end{array}}{\begin{array}{c} \text{Total}\;\text{number}\;\text{of}\;\text{consultations}\;\text{by}\\ \text{all}\;\text{specialties}\;\text{in}\;\text{the}\;\text{period} \end{array}}\;\right) \end{array}$$
  Or,
$$\text{Weight}\;\text{of}\;\text{vertex}\;\text{v}i\;=\;\frac{\begin{array}{c} \text{Total}\;\text{number}\;\text{of}\;\text{consultations}\;\text{by}\\ \text{physician}\;\text{v}i\;\text{in}\;\text{the}\;\text{period} \end{array}}{\begin{array}{c} \text{Total}\;\text{number}\;\text{of}\;\text{consultations}\;\text{by}\\ \text{all}\;\text{specialties}\;\text{in}\;\text{the}\;\text{period} \end{array}}$$ (1)
d) edge weight (Ew) between v*i* -> v*j*: represented by the ratio between the number of referrals from doctor v*i* to doctor v*j* and the total number of patients attended by doctor v*i*:
$$\text{Edge}\;\text{weight}\;\text{between}\;\text{v}i\;\text{-}>\;\text{v}j\;=\;\frac{\begin{array}{c} \text{Number}\;\text{of}\;\text{referrals}\;\left(\text{7-45}\;\text{days}\right)\;\text{from}\;\\ \text{physician}\;\text{v}i\;\text{to}\;\text{physician}\;\text{v}j\;\text{in}\;\text{the}\;\text{period} \end{array}}{\begin{array}{c} \text{Total}\;\text{number}\;\text{of}\;\text{patients}\;\text{attended}\;\\ \text{by}\;\text{physician}\;\text{v}i\;\text{in}\;\text{the}\;\text{period} \end{array}}$$ (2)

The network thus designed can be understood as a directed and weighted network. It is worth mentioning that, within this health system, the Amb-HCN has a basically self-regulated design, which may depend upon patient characteristics (such as place of residence, personal preferences, etc.), physician issues (such as specialty, location of practice, private network of collaboration and trust with other professionals, etc.), and terrain (such as availability of transportation, etc.). There are no referral flows between physicians explicitly promoted by the PHIP.

Several performance metrics were calculated at both the network and vertex levels. For the network, density, diameter, radius, average path length, global efficiency, clustering coefficient, and number of weak and strong components were calculated [16]. The calculation of the weighted versions of the metrics was prioritized, assigning the edges the weight Ew, as previously described. Unweighted versions were also calculated for some metrics for descriptive purposes or when the calculation of the weighted version was not applicable (Box 1).

**Box 1. Network-level performance metrics**

**Table:** 

**Metric**	**Definition**	**Practical interpretation**
Density	Ratio between the number of existing edges and the total number of possible edges in the graph.	Higher density suggests higher interconnection of vertices, implying stronger and more efficient communication or collaboration.
Diameter	Longest eccentricity (see Box 2) in the graph.	Both provide insights into the network’s overall size and reach. A large diameter or radius may indicate that information or influence can take a long time to spread across the network.
Radius	Shortest eccentricity (see Box 2) in the graph.	
Average path length	Average length (or distance) of the shortest paths between all pairs of vertices in the graph.	A shorter average path length indicates that information or influence can travel more quickly or efficiently within the network.
Global efficiency	Average of the inverse of the shortest distances between all pairs of vertices in the network.	Reflects how well the network maintains connectivity even when nodes or connections are removed. High values suggest network resilience, which is necessary for maintaining communication even in the face of disruptions or node failures.
Clustering coefficient	Closely related to the clustering coefficient of vertices (see Box 2), it is the ratio between the number of triangles (or closed triples of vertices) and the total number of triples (open and closed) in the graph.	Measures the degree to which nodes tend to cluster together. High clustering can indicate the presence of closely-knit subgroups, which can be advantageous for specialized collaboration or community building.
Weak components	Given a directed graph, a weakly connected component is a subgraph of the original graph where all vertices are connected to each other by some path, ignoring the direction of the edges.	Identifying weak and strong components can help understand the network’s overall connectedness and the presence of isolated subgroups. This information can be useful for optimizing communication and identifying key nodes.
Strong components	Given a directed graph, a strongly connected component is a subgraph of the original graph where all vertices are connected to each other by some path, respecting the direction of the edges.

For the vertices, the following measures were calculated: referrals made by the physician; referrals received by the physician; follow-up consultations performed by the physician; degree-in; degree-out; clustering coefficient; local efficiency; closeness-in; closeness-out; betweenness; eccentricity; PageRank (Google); subgraph centrality; Kleinberg’s authority score; Kleinberg’s HUB score; diversity [16]. The calculation of weighted versions of the metrics was prioritized, giving the edges the weight Ew, as previously described. Unweighted versions were also calculated for some metrics for descriptive purposes or when the calculation of the weighted version was not applicable (Box 2).

**Box 2. Vertex-level performance metrics**

**Table:** 

**Metric**	**Definition**	**Practical interpretation**	**Use in this study**
Referrals made by the physician	Number of referrals made by physician v*i* divided by the total number of patients seen by physician v*i*.	These related measures are intended to characterize: a) the strength of patient follow-up by the doctor, i.e., whether he or she practices a pattern of longitudinal follow-up or a more "episodic" or "fragmented" follow-up, where patients are seen on a sporadic or disconnected basis rather than consistently over an extended period of time; b) the extent to which the doctor shares patient care with other colleagues.	Candidate metrics for characterizing physicians in the “patient follow-up” dimension (see Data Analysis).
Referrals received by the physician	Number of referrals received by physician v*i* divided by the total number of patients seen by physician v*i*.		
Follow-up consultations performed by the physician	Number of follow-up consultations (after the patient’s first consultation with that physician) performed by physician v*i* divided by the total number of patients seen by physician v*i*.		
Degree-in	Number of edges that are incident on (or terminate at) vertex v*i*.	Indicate the extent to which a vertex receives (degree-in) and disseminates (degree-out) information (from or to immediate neighbors). Higher degree-in suggests that a node is well-connected and potentially influential. Higher degree-out indicates that a node is an information hub and can facilitate information flow.	
Degree-out	Number of edges that leave (or originate from) vertex v*i*.		
Closeness-in	Average number of steps required to reach vertex v*i* from all other vertices in the network.	Measure how close a node is to others in terms of incoming (closeness-in) and outgoing (closeness-out) paths. They indicate how quickly a node can access/gather information from the network (closeness-in) or disseminate/distribute information to the network (closeness-out).	Candidate metrics for characterizing physicians in the “centrality” dimension (see Data Analysis).
Closeness-out	Average number of steps required to reach all other vertices in the network starting from vertex v*i*.		
Betweenness	Number of shortest paths between all pairs of vertices in the network that pass through vertex v*i*.	It indicates the node’s potential to control or mediate information flow. Higher betweenness nodes act as information brokers and can control communication pathways.	
Eccentricity	Longest distance from (or to) vertex v*i* to (or from) the farthest vertex in the network, following the shortest paths.	Nodes with high eccentricity are relatively isolated and may require special attention to maintain their connectivity and participation in the network.	
PageRank (Google)	Measures the stationary probability that a given vertex v*i* will be visited, following a node-to-node weighted propagation scheme based on eigenvectors. Increases when several vertices point to vertex v*i* or when vertices with high rankings point to vertex v*i*.	Measures a node’s importance based on its connections and the importance of nodes linking to it. High PageRank nodes are considered influential, can have a significant impact on information dissemination, and may be important for targeted outreach.	
Subgraph centrality	Measures the number of subgraphs in which vertex v*i* participates (or closed arcs that originate in v*i*) and where longer arcs are exponentially down-weighted.	It can help identify nodes that are critical for the functioning of specific network subgroups, which is important for understanding niche roles within the larger network.	
Kleinberg’s authority score	These are essentially related measures that identify collections of densely connected vertices. Authorities are defined by a significant overlap of vertices that are densely pointed to by other vertices, called hubs. In turn, hubs densely point to several vertices with high authority scores. Hubs and authorities exhibit a mutual reinforcement relationship: a "good hub" is a vertex that points to many "good authorities," and a "good authority" is a vertex that is pointed to by many "good hubs".	These measures identify nodes that act as authorities (producers of content) or hubs (distributors of content) in a network. Authority nodes are content creators, while hub nodes facilitate distribution. This distinction is essential for optimizing content dissemination.	Characterizing physicians in the “relationship with authorities” dimension (see Data Analysis).
Kleinberg’s HUB score			
Clustering coefficient	Measures the proportion of vertices adjacent to vertex v*i* that are connected to each other.	High clustering indicates a node is within a tightly connected group. Higher clustering coefficients suggest that a node may play a key role in fostering collaboration and maintaining local cohesion within the network.	Descriptive purposes (see Data Analysis).
Local efficiency	For a given vertex v*i*, it is the average of the inverse distances between its adjacent vertices by traversing the shortest paths through the rest of the network (excluding vertex v*i*).	Measures how efficiently information or influence can spread from a node to its immediate neighbors. It reflects the node’s role in local information flow. Nodes with high local efficiency are effective at transmitting information within their local network neighborhoods.	
Diversity	Concept related to Shannon entropy, where high scores imply that a vertex distributes its connections to other vertices more evenly and equitably.	Measures how different a node’s connections are in terms of characteristics. Diverse nodes can bridge different communities and groups.

Because metrics from SNA have very widely different dimensionalities that would make numeric notation puzzling, their results were expressed in number of standard deviations above or below the mean of the analyzed group (i.e., the Z-score) to ease comparisons. However, for cluster analysis or any other statistical test, the original untransformed values were maintained.

Vertices that acted as articulation points (also called cut vertices) in the network were identified, defined as the vertices v*i* that, if removed, would increase the number of connected components in the graph or make a connected graph disconnected. Articulation points represent vulnerabilities for a connected network [16].

The vertex-level metrics (Box 2) were grouped into three dimensions (or constructs) according to the theoretical attributes they presumably reflect for the network and that are strategically significant from a managerial perspective. They are: a) **"patient follow-up profile"** dimension: aimed at characterizing the strength of patient follow-up by the doctor, i.e., whether he or she practices a pattern of longitudinal follow-up or a more "episodic" or "fragmented" follow-up; and, also, the extent to which the doctor shares patient care with other colleagues. Represented by the candidate metrics degree-in, degree-out, referrals made, referrals received, and follow-up consultations; b) **"relationship with authorities"** dimension: aimed at characterizing the degree to which each doctor achieves high authority scores and/or contributes to raising the authority score of other doctors to whom they refer patients. Represented by Kleinberg’s authority and HUB scores; c) **"centrality"** dimension: aimed at positioning the doctor relative to the network graph, assuming that the more central the position of a doctor in the network, the greater their ability to access and disseminate knowledge and information, or in other words, control the flow of information and influence the patient’s care trajectory. Represented by the candidate metrics closeness-in, closeness-out, betweenness, eccentricity, PageRank, and subgraph centrality.

To characterize each physician according to the three dimensions above, a cluster analysis of the vertex metrics within each construct was fitted using the non-hierarchical K-means technique, choosing the optimal number of clusters through visual inspection of graphs constructed by the average silhouette width and within-clusters sum of squares methods. Because there was a strong linear correlation (by Pearson’s *r*) among several candidate metrics within the above dimensions, some candidate variables were excluded from cluster analysis, though still allowing the inclusion of a closely correlated variable in the clustering (S1 Table). The resulting physician clusters were assigned labels that sought to be representative of the observed values of the vertex metrics within the clusters (see Table 3 in Results).

Medical communities detection was performed using the Infomap algorithm. This algorithm uses an information theoretic approach that is suitable for revealing community structures in weighted and directed networks. It uses the probability flow of random walks on a network as a proxy for information flows in the system and decomposes the network into modules by compressing the probability flow. A group of nodes among which information flows quickly and easily (in our case, more dense referrals and counter-referrals) can be aggregated and described as a single, well-connected module or community [16]. For this community detection procedure, edges and vertices were assigned the weights Ew and Vw, previously described.

Due to the strong imbalance in the number of consultations among physicians, a definition was established for "low consultation productivity" when a physician had performed less than 20% of the consultations expected for his or her specialty, according to the criterion below:

$$\begin{array}{c} \text{Total}\;\text{number}\;\text{of}\;\text{consultations}\;\text{by}\;\\ \text{physician}\;\text{v}i\;\text{in}\;\text{the}\;\text{period} \end{array}<\;\text{0.2}\;\text{x}\;\frac{\begin{array}{c} \text{Total}\;\text{number}\;\text{of}\;\text{consultations}\;\text{by}\;\\ \text{physician}\;\text{v}i’\text{s}\;\text{specialty}\;\text{in}\;\text{the}\;\text{period} \end{array}}{\begin{array}{c} \text{Total}\;\text{number}\;\text{of}\;\text{physicians}\;\text{in}\;\\ \text{physician}\;\text{v}i’\text{s}\;\text{specialty} \end{array}}$$ (3)

Doctors with low consultation productivity were included in the network-level SNA analysis but were excluded from the vertex-level performance analyses.

The statistical association of the newly created physician profiles (i.e., centrality, relationship with authorities and patient follow-up) was tested against each other as well as against the doctor’s specialty, the number of chronic comorbidities of the patients they cared for, and the medical communities they belonged to.

The association between categorical variables in contingency tables with dimensions greater than 2x2 was evaluated using the chi-square test and simple correspondence analysis with adjusted standardized residuals. Numerical variables were compared using the Kruskal-Wallis’ test, Mann-Whitney’s U test, or Student’s *t*-test, as appropriate. Correlations between numerical variables were evaluated using the Pearson’s (*r*) correlation coefficient.

The significance level was set at α = 0.05 (two-tailed). Whenever multiple comparisons were involved, the overall significance level was adjusted by means of the Bonferroni correction.

Social Network Analysis and clustering analyses were conducted using the igraph and factoextra packages of R 4.2.0 language (R Core Team (2022). R: A language and environment for statistical computing. R Foundation for Statistical Computing, Vienna, Austria. URL https://www.R-project.org/) in the RStudio environment (Posit team (2022). RStudio: Integrated Development Environment for R. Posit Software, PBC, Boston, MA. URL http://www.posit.co/). Other analyses, including data handling and pre-processing, were performed using Stata/SE 11.2 software (StataCorp LP, College Station, TX, USA).

The research was conducted following the principles of Brazilian ethical resolutions, particularly Resolution No. 466/12 of the National Health Council and its complementary resolutions. The project received approval from a research ethics committee endorsed by the National Commission for Ethics in Research (CONEP) (submission identifier No. 68241023.8.0000.5128. Collegiate decision No. 6.019.051).

## Results

### Network-level measures

During the study period, 666,263 individuals had at least one office visit, totaling 3,863,222 visits with one or more of the 4,554 physicians accredited by the PHIP. Only 15 physicians did not receive referrals or referred patients to other colleagues during the period and were excluded from SNA analyses. The results of the network-level measures are shown in Table 1.

**Table 1:** Results of the network-level measures.

Number of vertices	4,539
Number of edges	1,160,346
Weak components	1
Strong components	38
Clustering coefficient	0.255
Density	5.63%
Diameter	5 (unweighed); 34.417 (weighed)
Radius	3 (ignoring vertices with degree-out equal to zero)
Average path length	2.048 (unweighed); 3.346 (weighed)
Global efficiency	0.507 (unweighed); 0.339 (weighed)
Number of articulation point	27
Number of medical communities	15 (modularity = 0.149)

### Physicians with low consultation productivity

A total of 577 physicians (12.67%) were classified as having low consultation productivity (see Method). These physicians were responsible for only 18,058 referrals made (1.08%) and 17,961 referrals received (1.07%). The mean age of this group was 54.8 years old (95% CI = 53.7–55.8), not statistically different from that of physicians above this consultation threshold (55.5 years old, 95% CI = 55.2–55.8; t-test = 1.545, p = 0.123). Physicians with low consultation productivity had lower mean satisfaction scores than the rest [9.57 (95% CI = 9.51–9.64) vs. 9.65 (95% CI = 9.64–9.67), t-test = 3.92, p = 0.001].

The distribution of physicians according to consultation productivity and medical specialty is shown in S2 Table. The specialties most strongly associated with low consultation productivity were anesthesiology, general surgery, internal medicine, endoscopy, and family and community medicine.

### Vertex-level measures

All subsequent analysis of vertex-level metrics was conducted by excluding these 577 physicians with low consultation productivity, leaving 3,977 professionals. The distribution of vertex metric results showed strong variations among physicians, suggesting that those metrics may in fact be capturing different roles for each doctor in the network (Table 2).

**Table 2:** Results of the vertex-level measures.

	Number of standard deviations above or below the mean				
Measure	Minimum	25^th^ percentile	Median	75^th^ percentile	Maximum
Referrals made by the physician	-2.04	-0.56	-0.15	0.35	17.23
Referrals received by the physician	-2.65	-0.60	-0.10	0.42	16.08
Follow-up consultations performed by the physician	-1.37	-0.61	-0.23	0.29	10.45
Degree-in (unweighted)	-1.35	-0.76	-0.27	0.53	5.35
Degree-in (weighted)	-1.23	-0.70	-0.29	0.44	8.14
Degree-out (unweighted)	-1.32	-0.76	-0.27	0.53	5.40
Degree-out (weighted)	-2.04	-0.56	-0.15	0.35	17.23
Clustering coefficient (unweighted)	-3.03	-0.58	-0.08	0.49	7.79
Clustering coefficient (weighted)	-2.82	-0.61	-0.12	0.44	7.17
Local efficiency (unweighted)	-2.98	-0.53	-0.15	0.33	11.79
Local efficiency (weighted)	-1.08	-0.50	-0.37	-0.09	9.33
Closeness-in (unweighted)	-6.88	-0.53	0.03	0.62	3.70
Closeness-in (weighted)	-5.30	-0.69	-0.02	0.66	3.78
Closeness-out (unweighted)	-4.89	-0.57	0.02	0.63	3.82
Closeness-out (weighted)	-2.79	-0.69	-0.01	0.65	4.30
Betweenness (unweighted)	-0.62	-0.54	-0.36	0.13	13.12
Betweenness (weighted)	-0.41	-0.38	-0.28	-0.03	22.56
Eccentricity	-0.69	-0.69	-0.69	1.45	1.45
PageRank (Google)	-1.20	-0.70	-0.30	0.41	7.31
Subgraph centrality	-0.75	-0.65	-0.40	0.30	7.32
Kleinberg’s authority score	-0.09	-0.07	-0.05	-0.02	45.47
Kleinberg’s HUB score	-1.13	-0.50	-0.24	0.16	16.49
Diversity	-10.14	-0.28	0.26	0.61	1.28

### Cluster analysis for centrality, relationship with authorities and patient follow-up measures

The results of cluster analysis are reported in Table 3. Within the centrality dimension, the cluster analysis suggested the existence of three aggregates. Cluster 3 (with high PageRank scores, subgraph centrality, betweenness and closeness-in, and low eccentricity and closeness-out) was labeled as “central”. Cluster 2 (with low PageRank scores, subgraph centrality, betweenness and closeness-in, and high eccentricity and closeness-out) was labeled as “peripheral”. Cluster 1, with intermediate values, was labeled as “intermediate” (Table 3).

**Table 3:** Results of cluster analysis for centrality, relationship with authorities and patient follow-up measures, in number of standard deviations above or below the mean.

**Dimension: Centrality**									
**Cluster**	**n**	**Weighted closeness-out** ^(^ ^1^ ^)^	**Weighted closeness-in** ^(^ ^1^ ^)^	**Eccentricity** ^(^ ^1^ ^)^	**PageRank (Google)** ^(^ ^1^ ^)^	**Subgraph centrality** ^(^ ^2^ ^)^	**Unweighted betweenness** ^(^ ^2^ ^)^	**Weighted betweenness** ^(^ ^2^ ^)^	**Physician summary profile**
1	1,768	-0.1	-0.2	-0.7	-0.3	-0.3	-0.3	-0.1	Intermediate
2	1,258	+0.6	-0.6	+1.5	-0.7	-0.6	-0.5	0.0	Peripheral
3	951	-0.7	+1.1	-0.6	+1.4	+1.3	+1.2	+0.1	Central
**Dimension: Relationship with authorities**									
**Cluster**	**n**	**Kleinberg´s authority score** ^(^ ^1^ ^)^			**Kleinberg´s HUB score** ^(^ ^1^ ^)^				**Physician summary profile**
1	233	-0.04			+2.1				Seeks authorities (or “hubs”)
2	883	+0.15			+0.5				Is authority
3	2,828	-0.04			-0.4				Balanced
4	33	+0.10			+6.9				Seeks authorities (or “hubs”)
**Dimension: Patient follow-up profile**									
**Cluster**	**n**	**Referrals made** ^(^ ^1^ ^)^	**Referrals received** ^(^ ^1^ ^)^	**Follow-up appointments** ^(^ ^1^ ^)^	**Unweighted degree-in** ^(^ ^2^ ^)^	**Weighted degree-in** ^(^ ^2^ ^)^	**Unweighted degree-out** ^(^ ^2^ ^)^	**Weighted degree-out** ^(^ ^2^ ^)^	**Physician summary profile**
1	64	+4.7	+4.5	+4.4	+0.1	+0.2	+0.1	+4.7	Strong, shared
2	1,502	-0.6	-0.6	-0.6	-0.0	-0.1	-0.0	-0.6	Weak, shared
3	293	-0.9	-1.0	+1.2	-0.8	-0.5	-0.8	-0.9	Strong, prevalent
4	455	+1.3	+1.3	+1.1	+0.2	+0.3	+0.2	+1.3	Strong, shared
5	1,663	+0.2	+0.2	-0.1	+0.1	+0.1	+0.1	+0.2	Moderate, shared

^(1)^ Included in clustering analysis (see S1 Table for further details).

^(2)^ Not included in clustering analysis (see S1 Table for further details).

Regarding the dimension of relationship with authorities, K-means clustering suggested the existence of four cohesive groups. Cluster 2 (with the highest authority scores and moderate HUB scores) was labeled as “is authority”. Clusters 1 and 4 (with the highest HUB scores and intermediate authority scores) were labeled as “seeks authorities” or hubs. Cluster 3 (with intermediate scores) was labeled as “balanced” (Table 3).

Finally, as for the patient follow-up dimension, the cluster analysis suggested the existence of five groups. Clusters 1 and 4 had very high values of follow-up appointments, but also of referrals made and received. So, they were deemed to represent doctors with strong follow-up of patients, although shared with colleagues (labeled as “strong, shared” profile). Cluster 3 also had high patient follow-up rates, but very low rates of referrals made or received. So, it was deemed to represent doctors who assume most of their patients’ care (labeled as “strong, prevalent” profile). A similar rationale was used to label clusters 2 (“weak, shared” profile) and 5 (“moderate, shared” profile) (Table 3).

The distribution of physicians according to their profiles of centrality, relationship with authorities and patient follow-up by medical specialty is shown in Table 4. Cardiology, dermatology, endocrinology, ophthalmology, orthopedics, otolaryngology, pulmonology, psychiatry, and urology were strongly associated with the central profile. Surgical specialties predominated in the peripheral positions of the graph, along with clinical specialties such as nephrology, infectious diseases, internal medicine, and pediatrics.

**Table 4:** Distribution of physicians according to their profiles of centrality, relationship with authorities and patient follow-up by medical specialty.

Medical specialties^a^	n	Dimension									
		Centrality			Relationship with authorities			Patient follow-up			
Clinical		Central	Interme-diate	Peripheral	Balanced	Is authority	Seeks authority	Weak, shared	Moderate, shared	Strong, shared	Strong, prevalent
Cardiology	264	44.3% ↑^(1)^	35.2% ↓^(2)^	20.5% ↓^(1)^	58.3% ↓^(1)^	33.3% ↑^(1)^	8.3%	23.9% ↓^(1)^	61.7% ↑^(1)^	14%	0.4% ↓^(1)^
Dermatology	183	38.8% ↑^(1)^	50.8% ↑^(4)^	10.4% ↓^(1)^	76%	22.4%	1.6% ↓^(2)^	65.6% ↑^(1)^	33.9% ↓^(3)^	0.5% ↓^(1)^	0% ↓^(1)^
Psychiatry	102	37.3% ↑^(2)^	45.1%	17.6% ↓^(2)^	18.6% ↓^(1)^	53.9% ↑^(1)^	27.5% ↑^(1)^	1% ↓^(1)^	13.7% ↓^(1)^	85.3% ↑^(1)^	0% ↓^(2)^
Endocrinology and metabolism	140	48.6% ↑^(1)^	40%	11.4% ↓^(1)^	47.9% ↓^(1)^	40% ↑^(1)^	12.1% ↑^(2)^	7.1% ↓^(1)^	56.4% ↑^(1)^	36.4% ↑^(1)^	0% ↓^(2)^
Pulmonology	47	36.2% ↑^(3)^	38.3%	25.5%	55.3% ↓^(3)^	40.4% ↑^(2)^	4.3%	12.8% ↓^(1)^	76.6% ↑^(1)^	10.6%	0% ↓^(4)^
Allergy and immunology	31	32.3%	58.1%	9.7% ↓^(2)^	71%	29%	0%	32.3%	58.1% ↑^(4)^	9.7%	0%
Endoscopy	31	22.6%	29% ↓^(4)^	48.4% ↑^(3)^	77.4%	19.4%	3.2%	41.9%	54.8%	3.2%	0%
Nephrology	49	16.3%	34.7%	49% ↑^(2)^	36.7% ↓^(1)^	51% ↑^(1)^	12.2%	6.1% ↓^(1)^	53.1%	40.8% ↑^(1)^	0% ↓^(3)^
Rheumatology	37	24.3%	56.8%	18.9% ↓^(4)^	24.3% ↓^(1)^	51.4% ↑^(1)^	24.3% ↑^(1)^	0% ↓^(1)^	32.4%	67.6% ↑^(1)^	0% ↓^(4)^
Homeopathy	37	18.9%	59.5% ↑^(4)^	21.6%	43.2% ↓^(1)^	45.9% ↑^(1)^	10.8%	16.2% ↓^(2)^	32.4%	51.4% ↑^(1)^	0% ↓^(4)^
Geriatrics	36	11.1% ↓^(4)^	50%	38.9%	36.1% ↓^(1)^	58.3% ↑^(1)^	5.6%	2.8% ↓^(1)^	47.2%	50% ↑^(1)^	0% ↓^(4)^
Nutritional medicine	10	0% ↓^(4)^	60%	40%	40% ↓^(3)^	60% ↑^(2)^	0%	0% ↓^(3)^	50%	50% ↑^(2)^	0%
Neurology	57	19.3%	43.9%	36.8%	49.1% ↓^(1)^	38.6% ↑^(2)^	12.3%	17.5% ↓^(2)^	50.9%	31.6% ↑^(1)^	0% ↓^(3)^
Gastroenterology	67	28.4%	46.3%	25.4%	62.7%	34.3% ↑^(3)^	3%	20.9% ↓^(2)^	70.1% ↑^(1)^	9%	0% ↓^(3)^
Family and community medicine	21	9.5%	47.6%	42.9%	85.7%	0% ↓^(3)^	14.3%	85.7% ↑^(1)^	4.8% ↓^(2)^	0% ↓^(4)^	9.5%
Pediatrics	407	7.4% ↓ ^(1)^	43.2%	49.4% ↑^(1)^	95.8% ↑^(1)^	2% ↓^(1)^	2.2% ↓^(1)^	24.6% ↓^(1)^	8.6% ↓^(1)^	3.2% ↓^(1)^	63.6% ↑^(1)^
Internal medicine	337	18.7% ↓^(3)^	41.2%	40.1% ↑^(2)^	70.3%	24.3%	5.3%	46% ↑^(2)^	35.9% ↓^(3)^	17.8% ↑^(2)^	0.3% ↓^(1)^
Infectious diseases	20	5% ↓^(3)^	40%	55% ↑^(3)^	60%	40% ↑^(4)^	0%	20%	65% ↑^(3)^	15%	0%
Acupuncture	48	6.3% ↓^(2)^	56.3% ↑^(4)^	37.5%	35.4% ↓^(1)^	25%	39.6% ↑^(1)^	14.6% ↓^(2)^	29.2% ↓^(4)^	56.3% ↑^(1)^	0% ↓^(3)^
Hematology	34	8.8% ↓^(3)^	47.1%	44.1%	58.8%	35.3% ↑^(4)^	5.9%	0% ↓^(1)^	64.7% ↑^(2)^	35.3% ↑^(1)^	0% ↓^(4)^
**Mixed (clinical-surgical)**											
Otorhinolaryngology	140	37.1% ↑^(1)^	52.9% ↑^(3)^	10% ↓^(1)^	84.3% ↑^(1)^	12.9% ↓^(2)^	2.9% ↓^(4)^	66.4% ↑^(1)^	33.6% ↓^(3)^	0% ↓^(1)^	0% ↓^(2)^
Ophthalmology	292	37% ↑^(1)^	46.9%	16.1% ↓^(1)^	90.4% ↑^(1)^	9.6% ↓^(1)^	0% ↓^(1)^	87.3% ↑^(1)^	12.3% ↓^(1)^	0.3% ↓^(1)^	0% ↓^(1)^
Coloproctology	56	33.9% ↑^(4)^	35.7%	30.4%	67.9%	26.8%	5.4%	19.6% ↓^(2)^	76.8% ↑^(1)^	3.6% ↓^(3)^	0% ↓^(3)^
Angiology and vascular surgery	87	8% ↓^(1)^	49.4%	42.5% ↑^(3)^	72.4%	23%	4.6%	40.2%	57.5% ↑^(2)^	2.3% ↓^(2)^	0% ↓^(2)^
Gynecology and obstetrics	492	17.1% ↓^(1)^	54.7% ↑^(1)^	28.3% ↓^(4)^	78.3% ↑^(1)^	16.3% ↓^(2)^	5.5%	37.2%	49.6% ↑^(1)^	7.3% ↓^(1)^	5.9%
**Surgical**											
Orthopedics and traumatology	233	34.8% ↑^(1)^	50.2% ↑^(4)^	15% ↓^(1)^	66.1% ↓^(4)^	25.3%	8.6%	50.2% ↑^(1)^	45.1%	4.7% ↓^(1)^	0% ↓^(1)^
Urology	99	34.3% ↑^(3)^	45.5%	20.2% ↓^(3)^	79.8% ↑^(4)^	19.2%	1% ↓^(3)^	73.7% ↑^(1)^	26.3% ↓^(2)^	0% ↓^(1)^	0% ↓^(2)^
Hand surgery	15	20%	46.7%	33.3%	80%	20%	0%	40%	53.3%	6.7%	0%
Head and neck surgery	16	12.5%	43.8%	43.8%	75%	25%	0%	6.3% ↓^(2)^	81.3% ↑^(2)^	12.5%	0%
Mastology	53	32.1%	43.4%	24.5%	71.7%	22.6%	5.7%	28.3%	67.9% ↑^(1)^	3.8% ↓^(3)^	0% ↓^(3)^
Neurosurgery	54	16.7%	46.3%	37%	70.4%	22.2%	7.4%	5.6% ↓^(1)^	77.8% ↑^(1)^	16.7%	0% ↓^(3)^
General surgery	134	13.4% ↓^(2)^	29.9% ↓^(2)^	56.7% ↑^(1)^	69.4%	26.1%	4.5%	19.4% ↓^(1)^	64.9% ↑^(1)^	15.7%	0% ↓^(2)^
Cardiovascular surgery	29	3.4% ↓^(3)^	13.8% ↓^(2)^	82.8% ↑^(1)^	75.9%	17.2%	6.9%	20.7% ↓^(4)^	75.9% ↑^(1)^	3.4%	0%
Anesthesiology	145	13.8% ↓^(2)^	31% ↓^(2)^	55.2% ↑^(1)^	66.2%	16.6% ↓^(4)^	17.2% ↑^(1)^	55.2% ↑^(1)^	42.8%	2.1% ↓^(1)^	0% ↓^(2)^
Pediatric surgery	25	4% ↓^(3)^	16% ↓^(2)^	80% ↑^(1)^	100% ↑^(2)^	0% ↓^(2)^	0%	24%	56%	16%	4%
Plastic surgery	114	2.6% ↓^(1)^	42.1%	55.3% ↑^(1)^	79.8% ↑^(3)^	13.2% ↓^(3)^	7%	36%	56.1% ↑^(2)^	7.9% ↓^(4)^	0% ↓^(2)^
Thoracic surgery	13	0% ↓^(3)^	23.1%	76.9% ↑^(1)^	76.9%	7.7%	15.4%	38.5%	53.8%	7.7%	0%

Shaded values are significantly (p < 0.05) above (yellow) or below (dark-pink) the expected.

↑ = observed is above expected; ↓ = observed is below expected.

^(1)^ p < 0.001, ^(2)^ p < 0.01, ^(3)^ p < 0.05, ^(4)^ p < 0.1 [Significance level by adjusted standardized residual analysis (simple correspondence analysis)].

^a^ Some specialties were excluded due to low number of physicians (Genetics = 4; Physical medicine and rehabilitation = 9; Nuclear medicine = 2; Clinical neurophysiology = 2; Radiology and diagnostic imaging = 2; Radiation therapy = 7).

n = number of doctors.

There was a higher presence of doctors with an “is authority” profile in specialties such as cardiology, endocrinology, gastroenterology, geriatrics, homeopathy, nephrology, neurology, nutritional medicine, pulmonology, psychiatry, and rheumatology (Table 4).

The specialties significantly associated with strong and shared patient follow-up were acupuncture, internal medicine, endocrinology, geriatrics, hematology, homeopathy, nephrology, neurology, nutritional medicine, psychiatry, and rheumatology. Only pediatrics was significantly associated with strong and prevalent patient follow-up. There was a significant association between weak and shared follow-up for internal medicine and family medicine, among other specialties (Table 4).

### Associations between physician summary profiles

Table 5 reports pairwise comparisons among physician profiles within each dimension (i.e., centrality, relationship with authorities and patient follow-up). Physicians in a central position were more likely to have a strong and shared patient follow-up profile, while physicians in a peripheral position were associated with a strong and prevalent follow-up profile. Physicians in an intermediate position in the graph exhibited a non-statistically significant tendency to weak and shared patient follow-up.

**Table 5:** Associations between physician summary profiles.

Centrality	Relationship with authorities			Patient follow-up profile				Total
	Balanced	Is authority	Seeks authority	Weak, shared	Moderate, shared	Strong, shared	Strong, prevalent	
Central	572↓^(1)^	345↑^(1)^	34↓^(1)^	346	420↑^(4)^	162↑^(1)^	23↓^(1)^	**951 (23.9%)**
% of the line / % of the column	60.1% / 20.2%	36.3% / 39.1%	3.6% / 12.8%	36.4% / 23%	44.2% / 25.3%	17% / 31.2%	2.4% / 7.8%	
Intermediate	1,283↑^(4)^	351↓^(2)^	134↑^(3)^	695↑^(4)^	723	215	135	**1,768 (44.5%)**
% of the line / % of the column	72.6% / 45.4%	19.9% / 39.8%	7.6% / 50.4%	39.3% / 46.3%	40.9% / 43.5%	12.2% / 41.4%	7.6% / 46.1%	
Peripheral	973↑^(1)^	187↓^(1)^	98↑^(4)^	461	520	142↓^(3)^	135↑^(1)^	**1,258 (31.6%)**
% of the line / % of the column	77.3% / 34.4%	14.9% / 21.2%	7.8% / 36.8%	36.6% / 30.7%	41.3% / 31.3%	11.3% / 27.4%	10.7% / 46.1%	
**Relationship with authorities**								
Balanced				1,310↑^(1)^	1,109↓^(1)^	130↓^(1)^	279↑^(1)^	**2,828 (71.1%)**
% of the line / % of the column				46.3% / 87.2%	39.2% / 66.7%	4.6% / 25%	9.9% / 95.2%	
Is authority				153↓^(1)^	428↑^(1)^	295↑^(1)^	7↓^(1)^	**883 (22.2%)**
% of the line / % of the column				17.3% / 10.2%	48.5% / 25.7%	33.4% / 56.8%	0.8% / 2.4%	
Seeks authority				39↓^(1)^	126↑^(4)^	94↑^(1)^	7↓^(2)^	**266 (6.7%)**
% of the line / % of the column				14.7% / 2.6%	47.4% / 7.6%	35.3% / 18.1%	2.6% / 2.4%	
Total	**2,828 (71.1%)**	**883 (22.2%)**	**266 (6.7%)**	**1,502 (37.8%)**	**1,663 (41.8%)**	**519 (13.1%)**	**293 (7.4%)**	**3,977**

Shaded values are significantly (p < 0.05) above (yellow) or below (dark-pink) the expected.

↑ = observed is above expected; ↓ = observed is below expected.

^(1)^ p < 0.001, ^(2)^ p < 0.01, ^(3)^ p < 0.05, ^(4)^ p < 0.1 [Significance level by adjusted standardized residual analysis (simple correspondence analysis)].

Authorities were more likely to be in central positions within the network, while hub physicians were more often located in intermediate positions in the network, next to them. Physicians located in the periphery more frequently had a balanced relationship with authorities.

Authorities more frequently exhibited both strong and moderate shared patient follow-up profiles. Physicians with a tendency to seek authorities were more likely to have a strong and shared patient follow-up profile. Lastly, a weak follow-up profile was characteristic of physicians not classified as authorities or seeking authorities.

### Association of physician profiles with patient chronic comorbidities

Network physician profiles showed significant associations with features of the patients they treated, as assessed by the number of chronic comorbidities. These overall and within-specialties associations, along with significance tests, are reported in S3 Table.

Overall, physicians in central position (median number of chronic comorbidities per 100 patients = 192, p25-p75 = 141–247) or peripheral position (median = 189 per 100 patients, p25-p75 = 117–267) cared for patients with a higher number of chronic comorbidities as compared to doctors in intermediate positions in the graph (median = 177 per 100 patients, p25-p75 = 125–237). However, the association of physicians’ centrality profiles with the number of chronic diseases of their patients was less consistent across specialties, with distinctive patterns being observed for doctors from plastic surgery, internal medicine, and rheumatology (S3 Table).

Patients assisted by authority physicians (median = 224 per 100 patients, p25-p75 = 171–291) or hub physicians (median = 225 per 100 patients, p25-p75 = 167–291) had similar numbers of chronic comorbidities, significantly more than patients attended by physicians with a balanced relationship with authorities (median = 169 per 100 patients, p25-p75 = 115–229). This overall pattern also remained true across several specialties like allergy and immunology, cardiology, internal medicine, endocrinology, nephrology, ophthalmology, and orthopedics (S3 Table).

Amongst physicians with shared follow-up standards, the prevalence of chronic comorbidities in their patients increased stepwise as the intensity of patient follow-up increased (median = 160 per 100 patients, p25-p75 = 123–202 for weak follow-up; median = 217 per 100 patients, p25-p75 = 161–281 for moderate follow-up; and median = 249 per 100 patients, p25-p75 = 184–330 for strong patient follow-up). Patients cared for by physicians with strong and prevalent follow-up profile showed the lowest number of comorbidities (median = 38 per 100 patients, p25-p75 = 28–57). These same patterns were consistent across most doctor specialties (S3 Table).

### Articulation points

Twenty-seven physicians were identified as behaving as articulation points in the network, with the following characteristics: four cardiologists, three each of endocrinologists, ophthalmologists, and pediatricians, with the remaining 14 being in smaller numbers from other specialties. Ten doctors were in a central position in the network and 10 were in an intermediate position, 13 were authorities, and 14 had a moderate and shared patient follow-up profile.

### Medical communities

Overall, 15 medical communities were identified when considering the 4,539 physicians who received at least one referral or referred at least one patient to another colleague during the period (Table 1). Five of these communities were small clusters of physicians classified as having low consultation productivity (S2 Table). After excluding these, 10 communities remained. The number of doctors within each community, by medical specialty, is reported in S4 Table.

Box 3 reports the main properties of the medical communities detected. Emerging communities showed clear territorial segregation and tended to cluster in neighboring municipalities. The farther the networks were from Belo Horizonte (communities E, F and G), the more they tended to aggregate small conglomerates of doctors of primary, low-complexity specialties. Doctors from these communities were more likely to be peripheral in the graph, were seldom classified as authorities or hubs, and exhibited higher-than-expected patterns of strong and prevalent patient follow-up. Local networks that operated in Belo Horizonte and nearby municipalities (communities A, B, C and, to a lesser extent, D) exhibited separation of adult and pediatric specialties. Authorities and hub doctors were more concentrated in community A. As expected, communities with pediatric bias (B and D) had higher proportions of doctors which were peripheral in the graph and practiced a strong and prevalent patient follow-up profile. It is noteworthy that no community was detected with a primary seat in Contagem, the second most populous municipality in the area covered by our PHIP.

**Box 3. Characteristics of identified communities of physicians**

**Table:** 

**Medical community** ^a^	**Number of physicians (%)**	**% of total office visits**	**Description** ^b^	**Remarkable physician network profiles of centrality, relationship with authorities and patient follow-up** ^c^	**Territory of consultations** ^d^
Community A	3,288 (82.7%)	82.9%	Medical-surgical network without a strong bias towards specialties, but with a predominance of adult specialties	Lower proportion of doctors in peripheral position (28.6%); Moderately higher proportion of authorities (25.9%) or hubs (7.6%); Much lower ‘strong and prevalent’ patient follow-up (0.8%). Most doctors with ‘moderate and shared’ follow-up (46.3%).	89% Belo Horizonte; 6.2% Contagem (≈14.6 miles west); 3% Nova Lima (≈5.4 miles south); 1.8% other municipalities.
Community B	413 (10.4%)	8.6%	Medical-surgical network with a strong bias towards pediatric specialties	Higher proportion of doctors in peripheral position (46.7%); Much lower proportion of authorities (2.7%) or hubs (2.9%); Much higher ‘strong and prevalent’ patient follow-up (52.5%) and much lower for all other follow-up profiles.	87.3% Belo Horizonte; 7.2% Contagem (≈14.6 miles west); 4.8% Nova Lima (≈5.4 miles south); <1% other municipalities.
Community C	153 (3.8%)	5%	Medical-surgical network without a strong bias towards specialties, but with a predominance of adult specialties	Much higher proportion of doctors in central position (35.3%); Much lower proportion of authorities (9.8%) or hubs (2%); Much lower ‘strong and prevalent’ patient follow-up (3.3%).	82.2% Betim (≈20.6 miles west); 12.3% Belo Horizonte; 5.5% other municipalities.
Community D	31 (0.8%)	0.8%	Medical-surgical network with a strong bias towards pediatric specialties	Much higher proportion of doctors in peripheral position (58.1%); Absence of authorities or hubs (0%); Much higher proportion with ‘strong and prevalent’ patient follow-up (61.3%).	86.6% Betim (≈20.6 miles west); 5.3% Belo Horizonte; 1.7% Contagem (≈14.6 miles west); 4% Igarapé (≈30.1 miles southwest); 2.4% Esmeraldas (≈34.2 miles northwest).
Community E	36 (0.9%)	1.1%	Medical-surgical network, with a predominance of medium- and low-complexity clinical specialties	Much higher proportion of doctors in peripheral position (66.7%); Much lower proportion of authorities (5.6%) or hubs (0%); Higher ‘strong and prevalent’ (16.7%) or ‘weak and shared’ (66.7%) patient follow-up.	78.2% Pedro Leopoldo (≈26.6 miles north); 12.4% Matozinhos (≈31.7 miles north); 7% Belo Horizonte; 1.1% Ribeirão das Neves (≈21.1 miles northwest); 1.3% other municipalities.
Community F	34 (0.9%)	1.1%	Medical-surgical network, with a predominance of medium- and low-complexity medical specialties	Much higher proportion of doctors in peripheral position (52.9%); Much higher proportion of doctors with balanced relationship with authorities (88.2%); Higher proportion with ‘strong and prevalent’ patient follow-up (17.6%).	50.2% Lagoa Santa (≈25.4 miles north); 45.8% Vespasiano (≈20.3 miles north); 2.2% Belo Horizonte; 1.1% Matozinhos (≈31.7 miles north); 0.8% Pedro Leopoldo (≈26.6 miles north).
Community G	12 (0.3%)	0.2%	Medical-surgical network, with a predominance of medium- and low-complexity medical specialties	Much higher proportion of doctors in peripheral position (83.3%); Much higher proportion with ‘strong and prevalent’ patient follow-up (58.3%).	56% Santa Bárbara (≈67.7 miles east); 33.5% Barão de Cocais (≈61.1 miles east); 10.6% Belo Horizonte.
Community H	6 (0.2%)	0.1%	Small network of clinical pediatrics	NA	100% Santa Luzia (≈16.4 miles north)
Community I	2 (0.1%)	< 0.1%	Small network of 2 pediatricians	NA	100% Belo Horizonte
Community J	2 (0.1%)	< 0.1%	Small network of 2 doctors	NA	80% Nova Lima (≈5.4 miles south); 20% Belo Horizonte.

^a^ Medical communities were arbitrarily identified by letters with no specific meaning.

^b^ For further details, see S4 Table.

^c^ Values are percentages of physicians within each community. Reported percentages are significant at p < 0.05 [Significance level by adjusted standardized residual analysis (simple correspondence analysis)].

^d^ The locations are municipalities in the metropolitan region of Belo Horizonte. Data within parentheses represent approximate cardinal directions and distances for a standard car trip from a representative point in Belo Horizonte to a representative point in the destination municipality.

^NA^ Not analyzed due to low number of doctors.

## Discussion

A meaningful representation of the structure and organic functioning of an Amb-HCN involves understanding the roles and responsibilities of physicians during and after patient care in their offices. The presence of shared patients between two or more physicians reveals relationships that are established explicitly (i.e., established in contracts and formally monitored through performance indicators and value delivery), informally (i.e., established naturally and spontaneously, due to physician’s and patient’s preferences, sociodemographic characteristics, terrain, etc.), or even by chance, but represent a valuable source of information for the study of care networks [4, 9]. Physicians establish patient referral bonds more frequently with other physicians of the same sex and age group, when working in the same institution or geographically close, when completing their degree or residency training at the same educational institution, and when treating patients with similar clinical complexity, among other factors [17]. In any case, managing the functioning of an Amb-HCN presupposes the identification and measurement of these roles and responsibilities of the actors involved in direct patient care. Studies support the use of graph theory and SNA metrics to express the structure of healthcare networks, explain their care outcomes, study their changes over time, and observe how they react to the dynamic influences of central governance policies [9, 18, 19].

Studies suggest that various structural properties of healthcare networks may be associated with quality and safety of care [9, 20], although there remains a vast field of research to be explored. The dimensions used in this study to characterize the Amb-HCN were defined based on attributes considered strategically important for health policymakers [9, 20, 21]. The proposed analysis in this study aimed to identify the professionals who occupy prominent positions in the network, either due to their relationships with their peers, their connections with influential physicians, or because the topology of the network would change substantially without them. Thus, the following components were identified as key factors for identifying prominent professionals: patient referrals received from peers, relative importance in the network, and patient return behavior. Similar criteria were followed in another study [22].

Regarding the centrality dimension, it is assumed that doctors occupying more central positions in the graph have access to the most intense flow of information from colleagues who preceded them in patient care, and their own conduct can significantly influence the behavior of colleagues who will succeed them in patient care [7, 18, 20, 21]. Measures of centrality quantify the ability of a vertex to send, receive, or interrupt the flow of information [9]. Therefore, these doctors have a significant influence on the care journey of patients who seek them, even without conscious awareness or formally assuming this role [20, 21]. Another possible interpretation for the central role of some specialties is that they reflect the most prevalent nosology of the patient portfolio. Accordingly, in our setting, critical appraisal is required by the health manager in the face of the central position of doctors from specialties such as cardiology, endocrinology, pulmonology, orthopedics, and psychiatry. Of particular interest is the unexpected central position hold by urology doctors in our Amb-HCN, which may reflect the prevalent cultural practice in our setting of this professional assuming the health care of men in many situations. It may be argued that having many non-primary care doctors placed at the center of the network (as shown in Table 4) would not be the ideal structure for an Amb-HCN from the patient’s point of view, assuming that this position should be dominated by generalists and primary care physicians with the ability to coordinate patient care [10, 23]. Indeed, authors have proposed calculating the ratio between the centrality of primary care physicians and that of specialist physicians in the network [4, 23]. However, some studies have failed to demonstrate that Amb-HCN where primary care physicians were more central led to better health outcomes [23]. A study conducted with data from a private healthcare organization in Brazil found very similar centrality profiles to those of this study, with the most prevalent medical specialties being cardiology, endocrinology, dermatology, hematology, nephrology, orthopedics, and otorhinolaryngology [24]. Another analysis conducted in an Amb-HCN in the German public health system, where patients can seek care directly from specialists without needing to go through a primary care physician, also observed a notable dispersion of specialties involved in the care of patients with chronic diseases: 72% of the networks involved at least 10 distinct specialties, and the physicians with greater centrality in the networks were more often specialists (e.g., otorhinolaryngology, ophthalmology, etc.) [10]. On the other hand, peripheral positions in our Amb-HCN are predominantly occupied by surgical specialties, which can be readily explained by the nature of these specialties. However, the peripheral position of internal medicine, nephrology, and pediatrics is remarkable. In the case of the latter two, it could be explained by their strong profile of longitudinal patient follow-up (Table 4), which would lead them to assume a large part of patient care and have few connections with other colleagues. It is necessary to understand whether the unexpected strong peripheral presence of internal medicine can be explained by the same fact or, conversely, by the low care coordination role of a significant subgroup of these physicians, given the also prevalent weak longitudinal follow-up profile found in a large proportion of these professionals (Table 4) [2].

Regarding the profile of relationship with authorities, the convergence of authorities in certain medical specialties, in addition to reflecting the prevalent nosology of the patient portfolio, may indicate the concentration of referrals in few professionals considered qualified by their peers, and who keep their schedules more widely available for patient appointments [22]. Therefore, in our Amb-HCN, attention should be placed on evaluating access or qualification problems in specialties such as cardiology, endocrinology, gastroenterology, geriatrics, homeopathy, nephrology, neurology, nutritional medicine, pulmonology, psychiatry, and rheumatology. Also, the fact that some specialties concentrate physicians with a tendency to seek authorities may reflect the intrinsic clinical complexity of their own patient portfolios, a fact supported by the similar prevalence of chronic comorbidities among patients treated by these two categories (S3 Table). In the SNA approach that analyzes networks as mechanisms of social influence, studies suggest that physicians influence, and are influenced by, the behaviors and practices of colleagues with whom they are in closer contact, leading them to share similar clinical results [9, 21]. It may also be evidence of the tendency of clinical specialists who assume patient care to be knowledgeable about the other specialists that their patients seek [4], giving them greater authority to influence their choices. All of this justifies considering it possible that physicians classified as authorities and those who seek authorities (i.e., hubs) are a cohesive group, with shared patient portfolios and clinical practices.

The third profile proposed in this study aimed to reveal the patterns of patient follow-up by physicians, in light of the assumed responsibility of their specialty. Thus, just as it was evident the strong role of patient follow-up by specialists such as pediatrics, internal medicine, geriatrics, psychiatry, endocrinology, nephrology, and others, it was also evident the weak role of patient follow-up by significant subgroups of physicians from internal medicine, family medicine, and, to a lesser extent, pediatrics. In an Austrian Amb-HCN, where patient access to physicians was not restricted to primary care physicians as the entry point to the system, Sauter et al. [25] also demonstrated poor performance of family medicine and, to a lesser extent, internal medicine doctors, as coordinators of patient care, judging by the significant proportion of their patients who consulted with other physicians.

The analytical approach proposed in this study identified a wide dispersion of doctor profiles within specialties but was not designed to identify the specific drivers for. Doctor profile diversity within specialties may be informative from a managerial and policymaking point of view and instigates the adoption of a promising perspective of analysis: efforts should be made to understand to what extent the discrepancy between the practice of these physicians and the standards of their specialty is justified by specific areas of practice [10] or, in turn, explained by the physician’s lack of adherence to expected standards of patient care with quality and accessibility. Critically assessing each doctor’s profile in light of further administrative and clinical indicators may be elucidative.

Another relevant finding of this study is the significant association between network physician profiles (i.e., centrality, relationship with authorities and patient follow-up patterns) and the prevalence of chronic comorbidities among the patients they treated, even within specialties (S3 Table). This relationship may be bidirectional: patients with specific clinical characteristics may be more inclined to seek doctors with particular technical profiles or skills, and conversely, doctors with distinct technical profiles or skills may be more suited to attend to patients with specific clinical needs. As a result, they may be more likely capable of achieving better outcomes for their patients. This suggests, on the one hand, that for each patient’s health needs, it is possible and desirable to find the best combination of physician or Amb-HCN profiles that match best with those needs. On the other hand, it suggests that for each physician or Amb-HNC profile, it is possible and desirable to find patients with health needs that best fit those profiles [2, 9]. Patients cared for by authorities, hubs or doctors in central positions in the graph were those with the highest number of chronic comorbidities in this study. Actors in central positions in social networks tend to be considered opinion leaders and highly influential on the clinical decisions of colleagues [2, 9], a fact corroborated in this study, where authorities were more frequently central. We believe that the objective metrics of Amb-HCN proposed herein can be a valuable aid in identifying the compatibility (or incompatibility) between the patients’ health needs and the profile of their physicians or healthcare network, allowing healthcare managers to identify service gaps and steer solutions.

Although we did not investigate the relationship between network or doctor profiles and patient outcomes, the possibility that the physician’s position in the network, their relationship with authorities, and their patient follow-up profile are causally related to patients’ clinical outcomes should be considered by decision-makers and investigated in a timely manner. A study conducted in the Medicare population reported a significant positive association between the number of connections of primary care physicians with other physicians (i.e., degree) and healthcare costs, hospital admissions, days of hospitalization, admissions for primary care-sensitive conditions, emergency department visits, and specialist visits for patients under their care [18]. Another study also found that patients treated by physicians who shared care more intensely with other physicians had higher rates of hospitalization for primary care-sensitive conditions [23]. This can be interpreted as either arising from poor clinical coordination by the primary care physician or from a greater need for referral to specialists due to the higher clinical complexity of their patients.

The identification of different roles and responsibilities of physicians and specialties supports the theory that the health outcomes of individuals should be attributed not only to individual physicians but also to the functioning of the care network, collaboration, and information flow between physicians and specialties [17, 23, 24]. The demonstration of the natural emergence of self-organized communities of physicians (S4 Table and Box 3), with evident territorial and specialty segregation, reinforces this concept. This finding is a powerful management tool. Landon et al. [5] showed that naturally arranged communities of physicians around territories had professionals with close working relationships and were able to keep most hospitalizations (73%), emergency department visits (40%), primary care visits (88%), and specialist visits (60%) of patients within those networks boundaries. Networks and communities of professionals thus defined would be preferred targets of managers seeking physicians willing to become responsible for the health care of a defined patient population in capitation-based payment contracts [5, 26]. The territorial segregation of the communities identified in this study confirms the impression that regionalization is an important attribute of self-constituted Amb-HCN. In fact, in a recent study of the Amb-HCN of this same PHIP, we found that an extension of the Louvain community detection algorithm—which incorporated geographical information from medical offices—generated communities with more cohesive partitions that were territorially separated and stable over time [27]. The fact that no medical community with a predominant seat in Contagem municipality—the second most populous municipality in the PHIP coverage area—has emerged forces us to formulate the hypothesis that beneficiaries living in this municipality may need to seek care in nearby municipalities, such as Belo Horizonte or Betim, probably due to the insufficient specialty network in Contagem.

Several strengths of this study can be highlighted. By using administrative claim data routinely collected by the PHIP and by considering all more than 1 million beneficiaries of a healthcare plan that does not restrict the location or physician for consultations, the study has no missing data, avoided selection and response bias, and can be considered representative of a large population. This research incorporated some analytical approaches that are, at the very least, uncommon—to the best of our knowledge, unprecedented—for this type of study, such as the directional modeling of the network (commonly treated as non-directional by other authors) and the calculation of many measures and metrics grouped into constructs and aggregated into profiles by unsupervised methods, allowing the discovery of managerially relevant personas, whose external validity can be evaluated in other studies.

Some limitations of this study should also be acknowledged. As with any quantitative and cross-sectional representation of reality, the application of SNA certainly cannot capture all the complexity involved in the emerging relationships between actors. Part of the structural and functional topology observed in our Amb-HCN may originate from conjunctural, unstable, or seasonal factors that were not considered in the analysis. In addition, SNA requires multiple methodological choices appropriate for the study objectives but not necessarily relevant for all other purposes [2, 10]. Therefore, the extent to which the findings are reproducible and stable over time is unknown. Social Network Analysis results are known to be highly sensitive to design characteristics (e.g., directionality, definition and weighting of vertices and edges, etc.), size and scope of networks, evaluation metrics, inclusion or exclusion criteria for actors, and characteristics of health systems and geographic regions. Because each of these factors, the others held constant, can significantly impact the results of network metrics [17, 26], the generalizability of our findings to other contexts is not straightforward. Another limitation inherent to SNA is the influence of actors beyond the boundaries of the analyzed network. If the analysis did not include all relevant actors, it is unlikely that the reported results captured all the complexity of the Amb-HCN. Thirdly, because we used administrative claim data in which a patient visiting two doctors within 7–15 days may not necessarily represent an explicit and deliberate referral, some of the relationships found between physician may be spurious. An ideal approach to this problem, although methodologically complex, would be to restrict physician relationships to episodes of care or specific health problems of the patients, which would exclude circumstantial relationships between physicians who treat patients for unrelated health problems. This was the reason why this study considered consultations spaced by an interval between 7 and 45 days. Finally, although the analysis focused on the role of physicians as the main actors, it would be interesting to know to what extent the structure of the Amb-HCN depends on patient behaviors and preferences, the characteristics of the terrain, and civil transportation, among others.

## Conclusions

Viewing our Amb-HCN as a social network and applying measures based on graph theory and SNA provided emerging insights into the most influential actors and specialties, potential gaps in care, and the most prevalent diseases in our patient portfolio. The identification of self-constituted Amb-HCN can form a rational basis for developing more formal networks or monitoring patient care performance without assigning responsibility to a single physician. However, transferring research knowledge into actionable plans and decision-making by health authorities requires reflection, business expertise, and strategies based on continuous improvement cycles. The way network metrics reflect attributes of quality, access, and care coordination in healthcare is an evolving field. Defining operational metrics for the roles and responsibilities of healthcare professionals, understanding the functional structure of Amb-HCN, and evaluating their influence on patient health outcomes remain as challenges for researchers and health policymakers.

## Supporting information

S1 Table Pearson correlation coefficients between vertex-level measures. (DOCX)

S2 Table Distribution of physicians according to consultation productivity and medical specialty. (DOCX)

S3 Table Association between physician network profiles and the number of chronic comorbidities of the patients they cared for, by medical specialty. (DOCX)

S4 Table Number of physicians per identified community, according to their medical specialty. (DOCX)
