# Supplementary material for: Representation of the hierarchical and functional structure of an ambulatory network of medical consultations through Social Network Analysis, with an emphasis on the role of medical specialties
Source: PLoS One. 2024 Feb 15;19(2):e0290596. doi: 10.1371/journal.pone.0290596 (PMC10868750; doi:10.1371/journal.pone.0290596)
Supplement: S1 Table — (DOCX) [file pone.0290596.s001.docx]

| **S1 Table. Pearson correlation coefficients between vertex-level measures** | | | | | | | | | | | | | | | | | | | | | | | | | | |
| --- | --- | --- | --- | --- | --- | --- | --- | --- | --- | --- | --- | --- | --- | --- | --- | --- | --- | --- | --- | --- | --- | --- | --- | --- | --- | --- |
| Physi-cian profile dimen-sion | Weight-ed by Ew? | Present in final cluster-ing? | Measure | Refer-rals made by the physi-cian | Refer-rals received by the physi-cian | Follow-up consulta-tions performed by the physician | Degree-out (UnW) | Degree-out (W) | Degree-in (UnW) | Degree-in (W) | Author-ity score (W) | HUB score (W) | Close-ness-out (UnW) | Close-ness-out (W) | Close-ness-in (UnW) | Close-ness-in (W) | Between-ness (UnW) | Between-ness (W) | Eccentri-city | Page-Rank (Google) (W) | Sub-graph central-ity | Cluster-ing co-efficient (UnW) | Cluster-ing co-efficient (W) | Local effi-ciency (UnW) | Local effi-ciency (W) | Diver-sity (W) |
| Patient follow-up | NA | Yes | Referrals made by the physician | 1 |  |  |  |  |  |  |  |  |  |  |  |  |  |  |  |  |  |  |  |  |  |  |
|  | NA | Yes | Referrals received by the physician | 0,93 | 1 |  |  |  |  |  |  |  |  |  |  |  |  |  |  |  |  |  |  |  |  |  |
|  | NA | Yes | Follow-up consulta-tions performed by the physician | 0,66 | 0,63 | 1 |  |  |  |  |  |  |  |  |  |  |  |  |  |  |  |  |  |  |  |  |
|  | No | No | Degree-out | 0,14 | 0,10 | -0,01 | 1 |  |  |  |  |  |  |  |  |  |  |  |  |  |  |  |  |  |  |  |
|  | Yes | No | Degree-out | 1 | 0,93 | 0,66 | 0,14 | 1 |  |  |  |  |  |  |  |  |  |  |  |  |  |  |  |  |  |  |
|  | No | No | Degree-in | 0,14 | 0,12 | -0,02 | 0,99 | 0,14 | 1 |  |  |  |  |  |  |  |  |  |  |  |  |  |  |  |  |  |
|  | Yes | No | Degree-in | 0,13 | 0,14 | 0,07 | 0,93 | 0,13 | 0,94 | 1 |  |  |  |  |  |  |  |  |  |  |  |  |  |  |  |  |
|  |  |  |  |  |  |  |  |  |  |  |  |  |  |  |  |  |  |  |  |  |  |  |  |  |  |  |
| Relationship with authorities | Yes | Yes | Authority score | 0,02 | 0,03 | 0,03 | 0,06 | 0,02 | 0,06 | 0,10 | 1 |  |  |  |  |  |  |  |  |  |  |  |  |  |  |  |
|  | Yes | Yes | HUB score | 0,47 | 0,47 | 0,27 | 0,09 | 0,47 | 0,09 | 0,07 | 0,03 | 1 |  |  |  |  |  |  |  |  |  |  |  |  |  |  |
|  |  |  |  |  |  |  |  |  |  |  |  |  |  |  |  |  |  |  |  |  |  |  |  |  |  |  |
| Centrality | No | No | Closeness-out | 0,18 | 0,13 | -0,03 | 0,91 | 0,18 | 0,91 | 0,83 | 0,06 | 0,13 | 1 |  |  |  |  |  |  |  |  |  |  |  |  |  |
|  | Yes | Yes | Closeness-out | 0,41 | 0,43 | 0,41 | -0,60 | 0,41 | -0,59 | -0,46 | -0,03 | 0,16 | -0,64 | 1 |  |  |  |  |  |  |  |  |  |  |  |  |
|  | No | No | Closeness-in | 0,18 | 0,18 | -0,05 | 0,89 | 0,18 | 0,90 | 0,82 | 0,06 | 0,15 | 0,98 | -0,61 | 1 |  |  |  |  |  |  |  |  |  |  |  |
|  | Yes | Yes | Closeness-in | 0,19 | 0,24 | 0,20 | 0,64 | 0,19 | 0,66 | 0,75 | 0,06 | 0,07 | 0,60 | -0,17 | 0,61 | 1 |  |  |  |  |  |  |  |  |  |  |
|  | No | No | Between-ness | 0,04 | 0,03 | 0,00 | 0,87 | 0,04 | 0,87 | 0,90 | 0,06 | 0,02 | 0,72 | -0,43 | 0,70 | 0,58 | 1 |  |  |  |  |  |  |  |  |  |
|  | Yes | No | Between-ness | 0,40 | 0,43 | 0,38 | -0,04 | 0,40 | -0,03 | 0,12 | 0,01 | 0,14 | -0,06 | 0,43 | -0,04 | 0,35 | 0,01 | 1 |  |  |  |  |  |  |  |  |
|  | NA | Yes | Eccentri-city | -0,05 | -0,02 | -0,04 | -0,49 | -0,05 | -0,48 | -0,45 | -0,04 | -0,05 | -0,58 | 0,41 | -0,56 | -0,39 | -0,34 | 0,02 | 1 |  |  |  |  |  |  |  |
|  | Yes | Yes | PageRank (Google) | 0,13 | 0,12 | 0,05 | 0,94 | 0,13 | 0,95 | 0,98 | 0,06 | 0,06 | 0,84 | -0,49 | 0,83 | 0,75 | 0,90 | 0,09 | -0,45 | 1 |  |  |  |  |  |  |
|  | NA | No | Subgraph centrality | 0,11 | 0,08 | -0,03 | 0,96 | 0,11 | 0,96 | 0,89 | 0,05 | 0,08 | 0,82 | -0,53 | 0,80 | 0,56 | 0,89 | -0,05 | -0,38 | 0,91 | 1 |  |  |  |  |  |
|  |  |  |  |  |  |  |  |  |  |  |  |  |  |  |  |  |  |  |  |  |  |  |  |  |  |  |
| Not applicable (descriptive pourposes) | No | NA | Clustering coefficient | 0,04 | 0,01 | 0,00 | -0,40 | 0,04 | -0,42 | -0,48 | -0,04 | 0,01 | -0,39 | 0,18 | -0,40 | -0,47 | -0,44 | -0,09 | 0,29 | -0,45 | -0,34 | 1 |  |  |  |  |
|  | Yes | NA | Clustering coefficient | 0,06 | 0,02 | 0,07 | -0,49 | 0,06 | -0,50 | -0,53 | -0,09 | -0,02 | -0,51 | 0,31 | -0,53 | -0,42 | -0,46 | -0,01 | 0,35 | -0,47 | -0,42 | 0,89 | 1 |  |  |  |
|  | No | NA | Local efficiency | -0,05 | -0,08 | 0,00 | -0,31 | -0,05 | -0,32 | -0,33 | -0,04 | -0,06 | -0,33 | 0,10 | -0,36 | -0,30 | -0,31 | -0,05 | 0,21 | -0,30 | -0,26 | 0,87 | 0,87 | 1 |  |  |
|  | Yes | NA | Local efficiency | -0,18 | -0,17 | 0,16 | -0,36 | -0,18 | -0,36 | -0,22 | -0,03 | -0,22 | -0,55 | 0,35 | -0,56 | 0,02 | -0,19 | 0,18 | 0,26 | -0,23 | -0,32 | 0,18 | 0,39 | 0,41 | 1 |  |
|  | Yes | NA | Diversity | 0,08 | 0,02 | -0,10 | 0,05 | 0,08 | 0,04 | -0,19 | -0,16 | 0,07 | 0,16 | -0,20 | 0,14 | -0,31 | -0,09 | -0,31 | -0,07 | -0,10 | 0,04 | 0,14 | 0,13 | 0,04 | -0,32 | 1 |
| UnW = unweighted by Ew  W = weighted by Ew  Ew = weights attributed to the edges of the network  NA = not applicable | | | | | | | | | | | | | | | | | | | | | | | | | | |
