# Supplementary material for: Representation of the hierarchical and functional structure of an ambulatory network of medical consultations through Social Network Analysis, with an emphasis on the role of medical specialties
Source: PLoS One. 2024 Feb 15;19(2):e0290596. doi: 10.1371/journal.pone.0290596 (PMC10868750; doi:10.1371/journal.pone.0290596)
Supplement: S2 Table — (DOCX) [file pone.0290596.s002.docx]

| **S2 Table. Distribution of physicians according to consultation productivity and medical specialty** | | | |
| --- | --- | --- | --- |
|  | **Consultation productivity** | |  |
| **Medical specialty**^a^ | **Non-low** | **Low** | **Total** |
| Acupuncture | 48 (92%) | 4 (8%) | 52 |
| Allergy and immunology | 31 (100%)^↑(3)^ | 0 (0%)^↓(3)^ | 31 |
| Anesthesiology | 145 (76%)^↓(1)^ | 47 (24%)^↑(1)^ | 192 |
| Angiology and vascular surgery | 87 (91%) | 9 (9%) | 96 |
| Cardiology | 264 (85%) | 45 (15%) | 309 |
| Cardiovascular surgery | 29 (88%) | 4 (12%) | 33 |
| Hand surgery | 15 (94%) | 1 (6%) | 16 |
| Head and neck surgery | 16 (89%) | 2 (11%) | 18 |
| General surgery | 134 (77%)^↓(1)^ | 41 (23%)^↑(1)^ | 175 |
| Pediatric surgery | 25 (81%) | 6 (19%) | 31 |
| Plastic surgery | 114 (94%)^↑(3)^ | 7 (6%)^↓(3)^ | 121 |
| Thoracic surgery | 13 (81%) | 3 (19%) | 16 |
| Internal medicine | 337 (82%)^↓(1)^ | 76 (18%)^↑(1)^ | 413 |
| Coloproctology | 56 (97%)^↑(3)^ | 2 (3%)^↓(3)^ | 58 |
| Dermatology | 183 (95%)^↑(2)^ | 10 (5%)^↓(2)^ | 193 |
| Endocrinology and metabolism | 140 (88%) | 19 (12%) | 159 |
| Endoscopy | 31 (72%)^↓(2)^ | 12 (28%)^↑(2)^ | 43 |
| Gastroenterology | 67 (89%) | 8 (11%) | 75 |
| Geriatrics | 36 (86%) | 6 (14%) | 42 |
| Gynecology and obstetrics | 492 (88%) | 69 (12%) | 561 |
| Hematology | 34 (89%) | 4 (11%) | 38 |
| Homeopathy | 37 (88%) | 5 (12%) | 42 |
| Infectious diseases | 20 (87%) | 3 (13%) | 23 |
| Mastology | 53 (90%) | 6 (10%) | 59 |
| Family and community medicine | 21 (72%)^↓(3)^ | 8 (28%)^↑(3)^ | 29 |
| Nephrology | 49 (94%) | 3 (6%) | 52 |
| Neurosurgery | 54 (84%) | 10 (16%) | 64 |
| Neurology | 57 (81%) | 13 (19%) | 70 |
| Nutritional medicine | 10 (91%) | 1 (9%) | 11 |
| Ophthalmology | 292 (97%)^↑(1)^ | 8 (3%)^↓(1)^ | 300 |
| Orthopedics and traumatology | 233 (94%)^↑(2)^ | 16 (6%)^↓(2)^ | 249 |
| Otorhinolaryngology | 140 (89%) | 18 (11%) | 158 |
| Pediatrics | 407 (86%) | 66 (14%) | 473 |
| Pulmonology | 47 (80%)^↓(4)^ | 12 (20%)^↑(4)^ | 59 |
| Psychiatry | 102 (87%) | 15 (13%) | 117 |
| Rheumatology | 37 (90%) | 4 (10%) | 41 |
| Urology | 99 (91%) | 10 (9%) | 109 |
| **Total** | **3,977 (87%)** | **577 (13%)** | **4,554** |
| Shaded values are significantly (p < 0.05) above (yellow) or below (dark-pink) the expected.  ↑ = observed is above expected; ↓ = observed is below expected.  ^(1)^ p < 0.001, ^(2)^ p < 0.01, ^(3)^ p < 0.05, ^(4)^ p < 0.1 [Significance level by adjusted standardized residual analysis (simple correspondence analysis]  ^a^ Some specialties were excluded due to low number of physicians (Genetics = 4; Physical medicine and rehabilitation = 9; Nuclear medicine = 2; Clinical neurophysiology = 2; Radiology and diagnostic imaging = 2; Radiation therapy = 7) | | | |
