# Supplementary material for: Representation of the hierarchical and functional structure of an ambulatory network of medical consultations through Social Network Analysis, with an emphasis on the role of medical specialties
Source: PLoS One. 2024 Feb 15;19(2):e0290596. doi: 10.1371/journal.pone.0290596 (PMC10868750; doi:10.1371/journal.pone.0290596)
Supplement: S3 Table — (DOCX) [file pone.0290596.s003.docx]

| **S3 Table. Association between physician network profiles and the number of chronic comorbidities of the patients they cared for, by medical specialty** | | | | | | | | | | | | | |
| --- | --- | --- | --- | --- | --- | --- | --- | --- | --- | --- | --- | --- | --- |
|  | **Dimension** | | | | | | | | | | | | |
|  | **Centrality** | | | | **Relationship with authorities** | | | | **Patient follow-up** | | | | |
| **Medical specialties** | **Central**^a^ | **Interme-diate**^a^ | **Peripheral**^a^ | **p-value**^b^ | **Balanced**^a^ | **Is authority**^a^ | **Seeks authority^a^** | **p-value**^b^ | **Weak, shared**^a^ | **Moderate, shared**^a^ | **Strong, shared**^a^ | **Strong, prevalent**^a^ | **p-value**^b^ |
| Acupuncture | 155 | 184 | 179 | 0.758 | 176 | 162 | 190 | 0.257 | 146 | 184 | 190 |  | 0.063 |
| Allergy and immunology | 115 | 115 | 59 | 0.276 | 109 | 124 |  | 0.033 | 119 | 110 | 132^c^ |  | 0.034 |
| Anesthesiology | 288 | 267 | 255 | 0.148 | 261 | 278 | 268 | 0.170 | 260 | 267 | 387^d^ |  | 0.008 |
| Angiology and vascular surgery | 274 | 233 | 210 | 0.186 | 231 | 226 | 211 | 0.582 | 214 | 237 | 358 |  | 0.088 |
| Cardiology | 266 | 270 | 268 | 0.740 | 255^c^ | 286 | 300 | 0.003 | 207^d^ | 282 | 305 | 135^NC^ | <0.001 |
| Cardiovascular surgery | 450 | 423 | 492 | 0.717 | 422 | 508 | 550 | 0.311 | 378 | 487 | 450 |  | 0.686 |
| General surgery | 255 | 255 | 237 | 0.609 | 243 | 266 | 232 | 0.231 | 204^d^ | 249 | 285 |  | 0.002 |
| Pediatric surgery | 34 | 137 | 93 | 0.074 | 104 |  |  |  | 79 | 106 | 110 | 34 | 0.257 |
| Plastic surgery | 247 | 204 | 166^c^ | 0.004 | 185 | 201 | 191 | 0.555 | 144^d^ | 209 | 255 |  | <0.001 |
| Internal medicine | 187 | 194 | 207^d^ | 0.020 | 182^c^ | 228 | 205 | <0.001 | 172^e^ | 201^e^ | 393^e^ | 115^NC^ | <0.001 |
| Coloproctology | 233 | 218 | 232 | 0.145 | 230 | 230 | 214 | 0.903 | 176^c^ | 230 | 256 |  | 0.005 |
| Dermatology | 141 | 131 | 139 | 0.305 | 137 | 131 | 133 | 0.808 | 131^c^ | 142 | 154 |  | 0.045 |
| Endocrinology and metabolism | 224 | 217 | 234 | 0.749 | 207^c^ | 233 | 228 | 0.030 | 163^e^ | 212^e^ | 245^e^ |  | <0.001 |
| Endoscopy | 219 | 178 | 212 | 0.219 | 207 | 208 | 166 | 0.469 | 195 | 212 | 219 |  | 0.875 |
| Gastroenterology | 199 | 205 | 223 | 0.573 | 205 | 222 | 181 | 0.251 | 180^c^ | 211 | 241 |  | 0.018 |
| Geriatrics | 306 | 382 | 359 | 0.356 | 432 | 334 | 546 | 0.056 | 256 | 384 | 335 |  | 0.220 |
| Gynecology and obstetrics | 113 | 117 | 119 | 0.572 | 115 | 118 | 126 | 0.545 | 113^f^ | 124^f^ | 88^f^ | 84^f^ | <0.001 |
| Hematology | 261 | 322 | 352 | 0.059 | 322 | 328 | 374 | 0.322 |  | 318 | 349 |  | 0.021 |
| Homeopathy | 120 | 114 | 129 | 0.414 | 116 | 120 | 141 | 0.649 | 119 | 102 | 128 |  | 0.131 |
| Infectious diseases | 155 | 237 | 254 | 0.395 | 242 | 244 |  | 0.877 | 164 | 254 | 318 |  | 0.064 |
| Mastology | 192 | 203 | 184 | 0.842 | 196 | 214 | 176 | 0.393 | 201 | 190 | 288 |  | 0.088 |
| Nephrology | 401 | 385 | 368 | 0.945 | 317^c^ | 385 | 449 | 0.029 | 241^e^ | 343^e^ | 457^e^ |  | <0.001 |
| Neurosurgery | 284 | 352 | 324 | 0.060 | 315 | 373^c^ | 325 | 0.036 | 286 | 328 | 336 |  | 0.386 |
| Neurology | 274 | 307 | 288 | 0.562 | 292 | 277 | 290 | 0.906 | 271 | 281 | 294 |  | 0.702 |
| Ophthalmology | 158 | 166 | 178 | 0.197 | 159 | 204 |  | <0.001 | 157^c^ | 240 | 238 |  | <0.001 |
| Orthopedics and traumatology | 194 | 208 | 210 | 0.177 | 196^d^ | 222 | 225 | <0.001 | 193^e^ | 228^e^ | 262^e^ |  | <0.001 |
| Otorhinolaryngology | 163 | 161 | 178 | 0.414 | 166 | 161 | 138 | 0.749 | 153 | 188 |  |  | <0.001 |
| Pediatrics | 50 | 41 | 44 | 0.591 | 43 | 49 | 39 | 0.940 | 60^g^ | 118^g^ | 66^g^ | 36^g^ | <0.001 |
| Pulmonology | 318 | 318 | 316 | 0.558 | 318 | 297 | 366 | 0.263 | 248^c^ | 322 | 340 |  | 0.038 |
| Psychiatry | 189 | 200 | 235 | 0.114 | 220 | 192 | 222 | 0.293 | 114 | 209 | 202 |  | 0.240 |
| Rheumatology | 263 | 289^c^ | 244 | 0.029 | 297 | 274 | 265 | 0.373 |  | 263 | 282 |  | 0.218 |
| Urology | 211 | 193 | 190 | 0.198 | 194 | 205 | 191 | 0.774 | 188 | 245 |  |  | <0.001 |
| **Overall** |  |  |  |  |  |  |  |  |  |  |  |  |  |
| Median | 192 | 177^d^ | 189 | < 0.001 | 169^d^ | 224 | 225 | < 0.001 | 160^h^ | 217^h^ | 249^h^ | 38^h^ | < 0.001 |
| Percentile 25 | 141 | 125 | 117 |  | 115 | 171 | 167 |  | 123 | 161 | 184 | 28 |  |
| Percentile 75 | 247 | 237 | 267 |  | 229 | 291 | 291 |  | 202 | 281 | 330 | 57 |  |
| This table only reports medical specialties with 25 doctors or more.  ^NC^ This category was not considered for pairwise comparisons due to low number of physicians. ^a^ Values are median number of chronic comorbidities per 100 patients. ^b^ By Kruskal-Wallis’ test. ^c^ Values are statistically different (significance level < 0.05/2 = 0.025) from the other two categories jointly considered, and vice-versa. ^d^ Values are statistically different (significance level < 0.05/2 = 0.025) from the other two categories, and vice-versa. ^e^ Values are statistically different (significance level < 0.05/3 = 0.017) from the other two categories, and vice-versa. ^f^ Values are statistically different (significance level < 0.05/6 = 0.008) from the other categories, except for strong shared and strong prevalent patterns. ^g^ Values are statistically different (significance level < 0.05/6 = 0.008) from the other categories, except for weak shared and strong shared patterns. ^h^ Values are statistically different (significance level < 0.05/6 = 0.008) from all the other categories. All pairwise comparisons by Mann-Whitney’s test. | | | | | | | | | | | | | |
