# Supplementary material for: Representation of the hierarchical and functional structure of an ambulatory network of medical consultations through Social Network Analysis, with an emphasis on the role of medical specialties
Source: PLoS One. 2024 Feb 15;19(2):e0290596. doi: 10.1371/journal.pone.0290596 (PMC10868750; doi:10.1371/journal.pone.0290596)
Supplement: S4 Table — (DOCX) [file pone.0290596.s004.docx]

| **S4 Table. Number of physicians per identified community, according to their medical specialty** | | | | | | | | | | | |
| --- | --- | --- | --- | --- | --- | --- | --- | --- | --- | --- | --- |
|  | **Medical community** | | | | | | | | | |  |
| **Medical specialty**^a^ | **A** | **B** | **C** | **D** | **E** | **F** | **G** | **H** | **I** | **J** | **Total** |
| Acupuncture | 43 | - | 3 | - | 1 | 1 | - | - | - | - | 48 |
| Allergy and immunology | 25 | 4 | 1 | - | - | 1 | - | - | - | - | 31 |
| Anesthesiology | 141 | 1 | 2 | - | 1 | - | - | - | - | - | 145 |
| Angiology and vascular surgery | 82 | - | 3 | - | 2 | - | - | - | - | - | 87 |
| Cardiology | 245 | 2 | 11 | - | 4 | 1 | 1 | - | - | - | 264 |
| Cardiovascular surgery | 28 | - | 1 | - | - | - | - | - | - | - | 29 |
| Hand surgery | 15 | - | - | - | - | - | - | - | - | - | 15 |
| Head and neck surgery | 16 | - | - | - | - | - | - | - | - | - | 16 |
| General surgery | 126 | - | 6 | - | 2 | - | - | - | - | - | 134 |
| Pediatric surgery | - | 22 | - | 2 | 1 | - | - | - | - | - | 25 |
| Plastic surgery | 110 | - | 3 | - | - | 1 | - | - | - | - | 114 |
| Thoracic surgery | 12 | 1 | - | - | - | - | - | - | - | - | 13 |
| Internal medicine | 299 | - | 29 | - | 3 | 6 | - | - | - | - | 337 |
| Coloproctology | 55 | - | 1 | - | - | - | - | - | - | - | 56 |
| Dermatology | 174 | 1 | 6 | - | 1 | 1 | - | - | - | - | 183 |
| Endocrinology and metabolism | 131 | 1 | 5 | - | 2 | 1 | - | - | - | - | 140 |
| Endoscopy | 25 | 1 | 3 | - | - | 1 | 1 | - | - | - | 31 |
| Gastroenterology | 64 | - | 3 | - | - | - | - | - | - | - | 67 |
| Medical genetics | 1 | 3 | - | - | - | - | - | - | - | - | 4 |
| Geriatrics | 34 | - | 2 | - | - | - | - | - | - | - | 36 |
| Gynecology and obstetrics | 451 | - | 24 | - | 7 | 7 | 3 | - | - | - | 492 |
| Hematology | 33 | 1 | - | - | - | - | - | - | - | - | 34 |
| Homeopathy | 32 | 2 | 2 | - | - | 1 | - | - | - | - | 37 |
| Infectious diseases | 20 | - | - | - | - | - | - | - | - | - | 20 |
| Mastology | 52 | - | 1 | - | - | - | - | - | - | - | 53 |
| Family and community medicine | 18 | - | 2 | - | - | - | 1 | - | - | - | 21 |
| Physical medicine and rehab | 7 | - | - | - | - | - | - | - | - | - | 7 |
| Nephrology | 46 | 2 | 1 | - | - | - | - | - | - | - | 49 |
| Neurosurgery | 49 | 4 | 1 | - | - | - | - | - | - | - | 54 |
| Neurology | 52 | - | 3 | - | 1 | 1 | - | - | - | - | 57 |
| Nutritional medicine | 10 | - | - | - | - | - | - | - | - | - | 10 |
| Ophthalmology | 267 | 9 | 9 | - | 4 | 1 | 1 | - | - | 1 | 292 |
| Orthopedics and traumatology | 211 | 6 | 11 | - | 1 | 3 | 1 | - | - | - | 233 |
| Otorhinolaryngology | 124 | 5 | 6 | - | 2 | 1 | 2 | - | - | - | 140 |
| Pediatrics | 10 | 347 | 1 | 29 | 3 | 6 | 2 | 6 | 2 | 1 | 407 |
| Pulmonology | 42 | - | 4 | - | 1 | - | - | - | - | - | 47 |
| Psychiatry | 100 | 1 | 1 | - | - | - | - | - | - | - | 102 |
| Radiation therapy | 7 | - | - | - | - | - | - | - | - | - | 7 |
| Rheumatology | 37 | - | - | - | - | - | - | - | - | - | 37 |
| Urology | 90 | - | 8 | - | - | 1 | - | - | - | - | 99 |
| **Total** | **3,288** | **413** | **153** | **31** | **36** | **34** | **12** | **6** | **2** | **2** | **3,977** |
| ^a^ Some specialties were excluded due to low number of physicians (Nuclear medicine = 2; Clinical neurophysiology = 2; Radiology and diagnostic imaging = 2). | | | | | | | | | | | |
